# Effect of ocean acidification on the nutritional quality of marine phytoplankton for copepod reproduction

**DOI:** 10.1371/journal.pone.0217047

**Published:** 2019-05-20

**Authors:** Morgan T. Meyers, William P. Cochlan, Edward J. Carpenter, Wim J. Kimmerer

**Affiliations:** 1 Department of Botany, University of Otago, Dunedin, New Zealand; 2 Estuary and Ocean Science Center (formerly known as the Romberg Tiburon Center for Environmental Studies), San Francisco State University, Tiburon, CA, United States of America; Helmholtz-Zentrum fur Ozeanforschung Kiel, GERMANY

## Abstract

Phytoplankton are the oceans’ principal source of polyunsaturated fatty acids that support the growth and reproduction of consumers such as copepods. Previous studies have demonstrated ocean acidification (OA) can change the availability of polyunsaturated fatty acids to consumer diets which may affect consumer reproduction. Two laboratory experiments were conducted to examine the effects of feeding high-*p*CO_2_-reared phytoplankton on copepod egg production, hatching success, and naupliar survival. Marine phytoplankton *Rhodomonas salina*, *Skeletonema marinoi*, *Prorocentrum micans*, *and Isochrysis galbana* were exponentially grown in semi-continuous cultures at present (control) (400 ppm CO_2_, pH~8.1) and future (1,000 ppm CO_2_, pH~7.8) conditions and provided to *Acartia tonsa* copepods over 4 consecutive days as either nitrogen-limited (Exp. I) or nitrogen-depleted (Exp. II) mixed assemblage of phytoplankton. The composition of FAs in the phytoplankton diet was affected by *p*CO_2_ concentration and nitrogen deficiency; the ratio of essential fatty acids to total polyunsaturated fatty acids decreased in phytoplankton grown under high *p*CO_2_ and the mass of total fatty acids increased under nitrogen depletion. Additionally, total concentrations of essential fatty acids and polyunsaturated fatty acids in the diet mixtures were less under the high-*p*CO_2_ compared to the control-*p*CO_2_ treatments. Median egg production, hatching success, and naupliar survival were 48–52%, 4–87%, and 9–100% lower, respectively, in females fed high-*p*CO_2_ than females fed low-*p*CO_2_ phytoplankton, but this decrease in reproductive success was less severe when fed N-depleted, but fatty acid-rich cells. This study demonstrates that the effects of OA on the nutritional quality of phytoplankton (i.e., their cellular fatty acid composition and quota) were modified by the level of nitrogen deficiency and the resulting negative reproductive response of marine primary consumers.

## Introduction

Ocean acidification (OA) due to the increase in the partial pressure of CO_2_ (*p*CO_2_) is predicted to cause a decrease in ocean pH from 8.2 to 7.7 by the year 2100 [1,2]. Previous OA studies have demonstrated direct effects of this pH change on individual calcifying species such as corals [3] oysters [4] mussels [5] pteropods [6], and sea urchins [7]. Non-calcifying organisms such as crustaceans may respond to OA in ways that vary widely across taxonomic groups and that may be negligible or even beneficial for some species [8–10].

Copepods serve critical roles in marine ecosystems; they provide top down control on microplankton via grazing [11], stimulate nutrient recycling by producing fast-sinking fecal pellets [12,13], and are important prey items for higher trophic levels. Therefore, responses of copepods to OA could have significant consequences for ecosystem functioning, especially energy transfer and food web stability. Many copepods show resilience to high *p*CO_2_, even at extreme levels (7,000–10,000 ppm) [14–16], but the effect is species specific [17] and dependent on developmental stage [15,18,19]. Direct effects of OA conditions on adult copepods appear negligible or non-existent [14,17,20–25]. However, the general perception of copepods as impervious to OA is based largely on short-term studies of adult copepods, and thus may underestimate the impact of OA on future copepod populations [26,27]. Additionally, indirect effects of OA on copepods, such as those from OA-induced changes in trophic interactions, have been less-extensively studied yet are crucial to understanding the effects of OA on the reproductive success of copepods and their role in ecosystems.

OA conditions can alter the nutritional quality of phytoplankton as prey for consumers. Increased *p*CO_2_ can stimulate carbon fixation and may alter phytoplankton cellular stoichiometry in some phytoplankton species and assemblages [28,29]. The resulting high proportions of carbon relative to nitrogen and phosphorus render the phytoplankton cells nutritionally inferior for supporting growth of herbivore consumers [30–32]. High *p*CO_2_ may also decrease the essential fatty acid (EFA, S1 Table) content of phytoplankton and the relative contribution of polyunsaturated fatty acids (PUFA) to total fatty acids (FA), thus altering the nutritional quality of the phytoplankton for consumers [33,34], although some studies have found no effects of *p*CO_2_ on phytoplankton fatty acid profiles [35,36]. In a seminal laboratory experiment to assess the effects of OA on copepod development and reproduction, copepods provided with a single diatom species–*Thalassiosira pseudonana*—cultured under high *p*CO_2_ (~740 ppm) grew, developed, and reproduced more slowly than copepods fed a diatom diet produced under 380 ppm *p*CO_2_ [33]. Rossoll et al. [33] also found a negative correlation between *p*CO_2_ and cellular concentrations of PUFA in these marine phytoplankton. However, a similar study conducted with a diet of the dinoflagellate *Heterocapsa* sp., found no evidence for indirect effects of OA on copepod reproduction from a change in prey nutritional quality [37], suggesting that such indirect effects are prey-dependent. When community-level effects are investigated, phytoplankton FA concentrations and profiles are generally more influenced by phytoplankton community composition than *p*CO_2_ [38,39], and responses vary both as a function of location and in situ environment [38].

Changes in the nutritionally critical PUFA content of phytoplankton can have cascading effects throughout the food web because phytoplankton provide consumers with EFAs [40]. Animals require EFAs for health and reproduction, and EFAs must be obtained from their diet because animals cannot synthesize them *de novo* [41]. As consumers of phytoplankton in much of the ocean, copepods would be among the first to be affected by OA-induced changes in the nutritional quality of these essential macromolecules in their phytoplankton prey.

While the FAs in copepod triacylglycerides (TAGs) generally come from copepods’ recent phytoplankton diet, FAs in wax esters typically reflect their nutritional history over a longer period of time [42]. The EFAs and PUFAs found in TAGs are important for proper cell membrane structure and fluidity and serve as precursors for eicosanoid biochemicals, such as prostaglandins, that are critical signaling molecules for egg production and hatching [41]. FAs in wax esters are often invested into reproductive processes in adults [42] or selectively transferred to eggs to provide metabolic energy for naupliar development [42]. Lipids found in ovaries and other gonad tissues of crustaceans also contain higher proportions of omega-3 PUFAs, suggesting EFAs are likely a critical structural component for reproductive success [42]. Field studies have found positive correlations between EFAs and egg production rates in *Acartia tonsa* [43,44]. In laboratory studies, reproductive capacities, such as egg production and hatching success, decrease under diets with limited EFAs for *A*. *tonsa* [45,46] and for other copepod species [47–50].

While evidence suggests direct effects of OA on copepods are negligible [14,17,20–23,25], reproduction of copepods still may be indirectly affected by OA-induced changes in the EFA content of dietary phytoplankton. Adult female copepods fed a high-*p*CO_2_ phytoplankton diet produced fewer eggs, and recruitment of nauplii was lower than those in an ambient control [33,51], whereas other studies have found no evidence of indirect dietary effects on copepod reproduction [37]. However, few studies on this topic have been published to date, and most have used only a single prey species of phytoplankton [33,37]. Recently it has been reported that increased *p*CO_2_ may alter the nutrient flow among various phytoplankton groups resulting in differential growth rates, changes in the stoichiometry (particulate C: particulate N ratios and particulate C: particulate P ratios) and possible propagation through the food-web, leading to potentially negative impacts on consumers [52 and references therein]. Such changes in macronutrient availability, specifically inorganic nitrogen, in association with alterations in OA were investigated here since N is generally considered the limiting macronutrient in most coastal marine systems (e.g., [53,54]), and its availability and resulting impact on phytoplankton growth are known to affect the production and composition of fatty acids by phytoplankton [55, 56–59].

Because direct effects of OA on copepod reproduction are unlikely [e.g.,14,18] and most coastal ecosystems are N-limited, our study focused on potential indirect effects of OA on copepod reproduction through OA-induced changes in their diet within the context of N-limited and N-depleted environments. Specifically, in this study we assessed 1) the impact of increased *p*CO_2_ on the EFA composition of four marine phytoplankton species: *Rhodomonas salina*, *Skeletonema marinoi*, *Prorocentrum micans*, and *Isochrysis galbana*, and 2) the effect of a dietary mixture of these phytoplankton on the reproductive success of the particle-feeding copepod *Acartia tonsa*. These assessments were carried out using phytoplankton at two levels of N deficiency (N-limited and N-depleted) to determine whether changes in the nutritional quality of phytoplankton (i.e., their cellular FA composition and quota) expected from *p*CO_2_ were modified by the level of N deficiency, and whether such changes (if any) affected copepod reproductive success. Based on previously published studies, we expected phytoplankton EFA compositions to be lower under high-*p*CO_2_ conditions, and for the copepods fed a diet of phytoplankton grown under high-*p*CO_2_ conditions to produce fewer eggs with lower hatching success and naupliar survival rates than copepods fed phytoplankton maintained at present-day (control) *p*CO_2_ conditions.

## Method

### Experimental design

The four phytoplankton species were grown separately as individual unialgal cultures under control (400 ppm) and high (1,000 ppm)-*p*CO_2_ concentrations to serve as treatment diets to freshly collected copepods. For each *p*CO_2_ treatment, three or four phytoplankton species of each treatment were mixed together and fed to copepods over the course of four days after which egg production, hatching success, and naupliar survival were measured. Exponentially-grown unialgal, but not axenic, phytoplankton cultures were maintained under virtually identical conditions during this study, but provided to the copepods at two different levels of N deficiency. In Experiment I (December 2014), the cells were harvested daily for four days from the semi-continuous cultures when the external concentrations of nitrate plus nitrite [(NO_3_^-^ + NO_2_^-^) hereafter referred to as nitrate (NO_3_^-^)] approached levels considered limiting to phytoplankton uptake and growth, whereas in Experiment II (June 2015), the external concentrations of NO_3_^-^ in the cultures were routinely at or below the limit of analytical detection (0.04 μM; see S2 Table) when harvested. These two levels of N deficiency are considered here as N-limited (Exp. I) and N-depleted (Exp. II), however based on the phytoplankton specific growth rates estimated from daily sampling of relative fluorescence, all phytoplankton species were maintaining exponential growth rates (data not shown) when initially provided as prey to the copepods during both experiments.

The marine phytoplankton species used as food sources for copepods were the haptophyte *Isochrysis galbana* (ISO; UTEX LB987 isolated from Port Erin, Isle of Man), the dinoflagellate *Prorocentrum micans* (PRO; CCMP689 isolated from La Jolla, California, USA), the cryptophyte *Rhodomonas salina* (RHO; CCMP1319 isolated from Long Island Sound, USA), and the diatom *Skeletonema marinoi* (SKE; UTEX LB2308 isolated from Galveston, Texas, USA). These species were chosen because they are rich sources of EFA [60–64,39] and are readily eaten by *Acartia tonsa*.

We collected adult *A*. *tonsa* using a surface plankton tow (202-μm mesh, 30-cm diameter) from San Francisco Bay conducted from the pier of the Estuary & Ocean Science Center (37° 53’ 28” N, 122° 26’ 47” W). Copepods were collected between 0800 and 1000 h local time on the day before the experiment started, and *A*. *tonsa* was immediately hand-picked from the rest of the zooplankton community under a light microscope using a wide-mouth plastic pipette. For both collections (2014 and 2015), *A*. *tonsa* dominated the mesozooplankton community and were relatively easy to distinguish from other copepods, although it is possible individuals of other *Acartia* species may have been in the samples. Immediately after picking, *A*. *tonsa* individuals were sorted evenly (50 copepods per bottle, 25 males and 25 females) into two polycarbonate bottles each containing 1-L filtered (0.2-**μm**, Whatman Polycap TC capsule filter 91372B) natural seawater collected in 2010 from Half Moon Bay, CA (37° 29’ 31”N, 122° 30’ 02” W) that was stored in a dark tank at ambient outdoor temperature until use. A density of 50 copepods L^-1^ was used to ensure fertilization for the assessment of reproductive success at the end of the experiment. Copepods were maintained at 20°C on a 16:8 h light: dark cycle and the water was gently aerated. We did not provide copepods with food until the experiment began the following morning.

### Phytoplankton culturing

Unialgal cultures of the four phytoplankton species were grown separately in 1.8-L polycarbonate bottles at 20°C under continuous light (180 μmol photons m^-2^ s^-1^) provided by a series of linear fluorescent bulbs (Vita-Brite Enviro-Lume F32T8). The basal medium consisted of nutrient-poor, natural seawater previously collected from Half Moon Bay, CA in 2008 and stored in the dark since collection. This basal medium was enriched with macronutrients, vitamins and trace metals to achieve *f*/2 concentrations [65] as outlined by [66], but with nitrogen added as nitrate (NaNO_3_) to a final concentration of 50 μM, rather than 886 μM, to reduce the potential phytoplankton biomass yield, and to ensure that N was the growth-limiting nutrient. All other *f*/2 enrichments were provided at concentrations considered saturating for phytoplankton growth. The enriched seawater medium was then sterile-filtered (0.2-μm Whatman PolyCap TC capsule filter 91372B) prior to its use, and although septic techniques were used throughout the culturing process to minimize fungal growth and bacterial contamination, cultures should be considered unialgal, not axenic.

**Fig 1:**
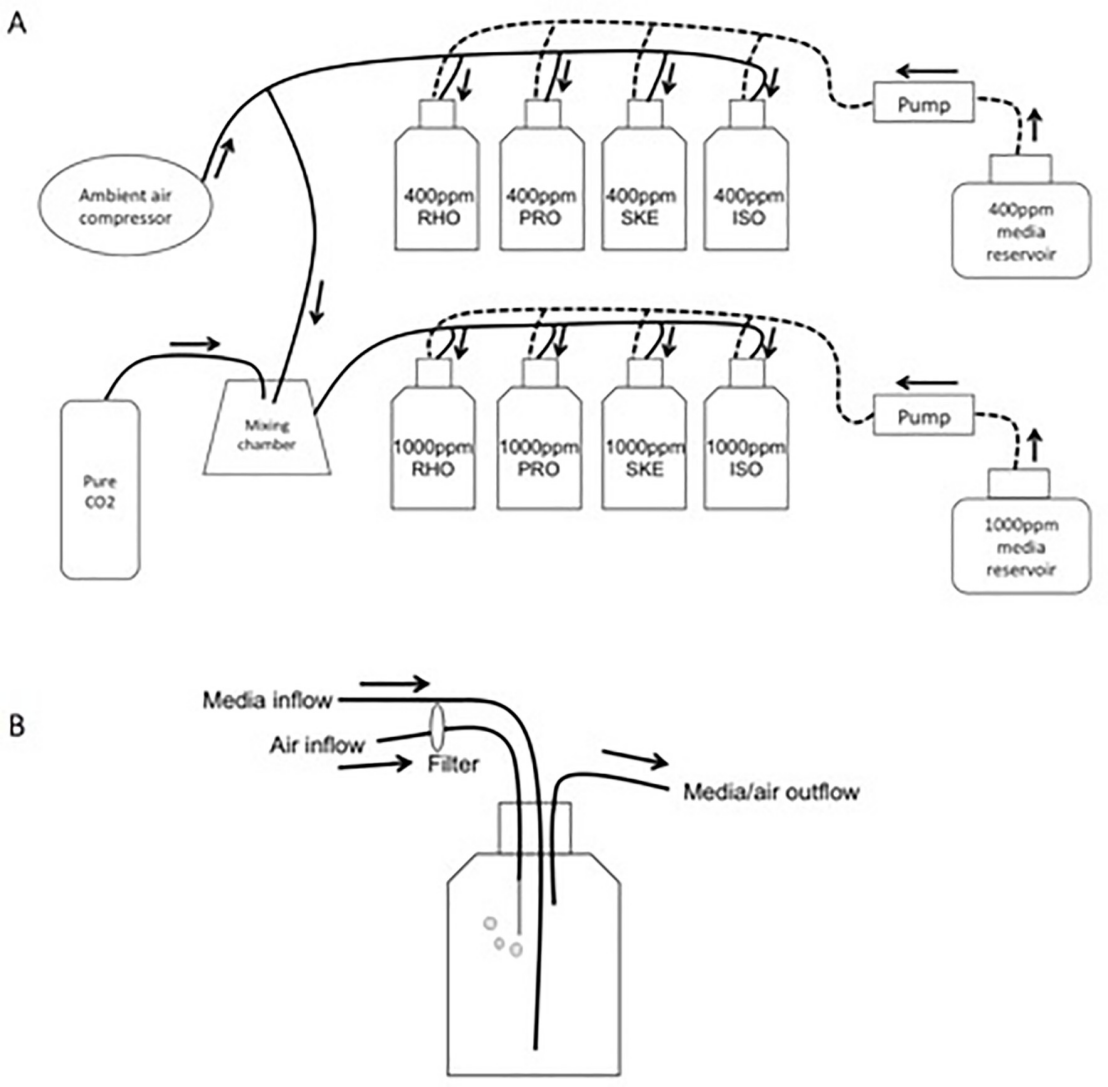
Diagram of experimental configuration. A: Ambient air at 400 ppm CO_2_ was bubbled into four 2-L phytoplankton culture bottles for the control *p*CO_2_ treatment, and ambient air mixed with pure CO_2_ to a final concentration of 1,000 ppm CO_2_ was bubbled into another four 2-L phytoplankton culture bottles for the high-*p*CO_2_ treatment; medium was delivered to each bottle from pre-bubbled 20-L media reservoirs. B: Each semi-continuous culture bottle had ports for medium inflow, gas inflow, and media/gas outflow.

Each phytoplankton culture was acclimated to the respective *p*CO_2_ treatment for a minimum of five generations of batch growth before implementing semi-continuous culturing to maintain cells in exponential growth. Cultures were diluted daily with fresh culture medium previously adjusted to the appropriate temperature and *p*CO_2_ using Gilson MiniPuls Evolution peristaltic pumps (Fig 1). The culture volume removed each day (17% of the container volume) was used for analysis of cellular fatty acid (FA) concentrations and to feed the copepods over a series of four days. The use of continuous 24 h light eliminated potential cellular FA variability due to diel variations in nitrogen uptake and growth rates [67].

### pCO_2_ treatments and feeding regime

Cultures of each phytoplankton species were divided into two parts and aerated with either control (400 ppm) or high (1,000 ppm) *p*CO_2_ (Fig 1). Ambient air (400 ppm) was used as the control-*p*CO_2_ treatment gas. The 1,000 ppm CO_2_ gas was made by mixing ambient air with pure CO_2_ gas (Praxair carbon dioxide UN#1013). Treatment gas flowed through an air filter (Whatman Vacu-guard L#5480) and then entered the culture bottle through a glass capillary tube attached to the end of silicon tubing. *p*CO_2_ conditions were monitored continuously using Li-Cor infrared gas analyzers (LI-280, LiCor, Lincoln, NE, USA). Over the course of an experiment, the control-CO_2_ air ranged from 370 ppm to 422 ppm and the high-CO_2_ gas ranged from 986 ppm to 1,048 ppm (accuracy < 3% of reading).

Phytoplankton of all four species, harvested daily from the semi-continuous cultures, were mixed together and fed daily to adult *A*. *tonsa* for four consecutive days in 1-L feeding chambers (50 copepods L^-1^) at the respective *p*CO_2_ level. Control phytoplankton mixtures were fed to copepods kept in seawater medium bubbled with 400 ppm *p*CO_2_, and high-*p*CO_2_ phytoplankton mixtures were fed to copepods kept in seawater medium bubbled with 1,000 ppm *p*CO_2_. Before each feeding event, copepod water was replaced with fresh seawater that had been pre-bubbled for at least 24 h with the appropriate CO_2_ concentration. Cross treatments (i.e., control phytoplankton fed to high-*p*CO_2_ copepods and high-*p*CO_2_ phytoplankton fed to control copepods) were not implemented because phytoplankton can rapidly change their FA content when transferred to a new *p*CO_2_ environment [33]. The feeding diet was composed of a balanced carbon-equivalent mixture of the phytoplankton species such that the total of 600 μg carbon provided daily to each 1-L feeding chamber was evenly divided among the phytoplankton species (200 μg carbon each of *R*. *salina*, *S*. *marinoi* and *P*. *micans* during Experiment I, and 150 μg carbon each of *R*. *salina*, *S*. *marinoi*, *P*. *micans* and *I*. *galbana* during Experiment II). The culture volumes harvested of each species were based on daily determination of cell density using microscopy (Zeiss Axioskop microscope) and species-specific cellular carbon content from the literature. Carbon content of each species used for these determinations were: *R*. *salina*, 2.9 x 10^−5^ μg C cell^-1^ [63], *S*. *marinoi*, 3.0 x 10^−5^ μg C cell^-1^ [68], *P*. *micans*, 2.7 x 10^−3^ μg C cell^-1^ [69], and *I*. *galbana*, 8.8 x 10^−6^ μg C cell^-1^ [70]. Based on these calculations, 1–10% of each unialgal culture was harvested daily for the feeding mixtures, and between 5.28 x 10^4^ and 6.71 x 10^6^ cells of each species were fed to copepods, depending on phytoplankton species, unialgal culture density, and experiment (Table 1).

**Table 1:** Phytoplankton feeding mixtures and copepod sample size.

	Species	Experiment 1		Experiment II	
		400 ppm	1,000 ppm	400 ppm	1,000 ppm
**Number of cells fed to copepods on Day 4**	*R*. *salina*	6.70x10^6^	6.71x10^6^	5.00 x10^6^	5.07 x10^6^
	*S*. *marinoi*	6.67x10^6^	6.50 x10^6^	4.93 x10^6^	4.92 x10^6^
	*P*. *micans*	7.31x10^4^	7.32 x10^4^	5.28 x10^4^	5.50 x10^4^
	*I*. *galbana*	0	0	1.83x10^7^	1.87 x10^7^
**Number of viable female copepods per *p*CO**_**2**_ **treatment at time of egg collection**		6		16	

Abundance and species composition of the phytoplankton feeding mixtures, and the copepod sample size (n) for Experiments I and II performed in December 2014 and June 2015, respectively.

After each daily seawater exchange and feeding event, the culture containers were placed on a rotating plankton wheel to ensure complete mixing of food and maintained for 24 h on a 16:8 h L:D cycle at 20°C. The pH of each phytoplankton feeding mixture and copepod pre-bubbled seawater was measured before and after each feeding event (S1 Table). We measured pH with a pH meter (Denver instruments model 215) equipped with a glass electrode (Scientific Orion 8102BNUWP) and calibrated with NBS buffers (Fisher Scientific Cat. No. SB105).

Before the final feeding event on Day 4, each individual female was removed from the 1-L container using a wide-mouth plastic pipette and gently transferred to a smaller, 80-mL polycarbonate container (one female per container) so we could track reproductive success independently for each individual female copepod (12 containers in Experiment 1, 6 per treatment; 32 containers in Experiment II, 16 per treatment). Each of the smaller containers were then placed on the rotating plankton wheel for the final 24-h feeding period.

### Assessment of copepod reproductive success

On the fifth day after the final 24-h feeding period, we gently poured the contents of each 80-mL container (one female copepod and any eggs produced) into a plastic petri dish (one petri dish per container). We used a glass pipette to collect all eggs produced by each *A*. *tonsa* female and distributed them among well plates in 2.5-mL of untreated seawater (one egg per well). Each female’s eggs were counted and distributed in well plates separately from those of the other females, and counts were recorded as eggs produced female^-1^. Each egg was then observed using a light microscope every five hours for three days to record hatching success and naupliar survival. Successful hatching was defined by a nauplius fully emerging from the egg casing, whereas naupliar survival was defined by its ability to move and respond normally to a gentle touch stimulus from a glass pipette.

### Nutrient and fatty acid analyses

Samples (17 mL) collected for nitrate analysis from phytoplankton cultures were filtered through combusted (450°C for 6 h) Whatman GF/F filters into 20-mL plastic vials and frozen until the day before analysis. The samples were analyzed with a Bran + Luebbe Technicon-II Auto Analyzer using a standard Cd reduction technique [71].

Phytoplankton samples (100 mL) for fatty acid methyl ether (FAME) analysis were collected on the fourth day from each unialgal culture at both *p*CO_2_ treatments, and were extracted wet via microwave-assisted solvent extraction (MASE; MARS-5 system) with 2:1 dichloromethane:methanol (DCM:MeOH) and the addition of nondecanoic (C19:0) fatty acid as the internal standard following methods of [72]. The total lipid extract was filtered through combusted, solvent-rinsed glass wool to remove particulates; evaporated to dryness and re-dissolved in the original 2:1 DCM:MeOH solvent mixture. Base hydrolysis of extract used 0.1N KOH to isolate the polar fraction containing fatty acids which were converted into their corresponding methyl esters using boron trifluoride (10% in methanol). FAMEs were quantified with an Agilent 6890N gas chromatograph with flame ionization detector (GC-FID) and identified with an Agilent 6890 gas chromatograph coupled to an Agilent 5975 mass spectrometer (GC-MS). Both instruments utilized a 60 m DB5-MS column. For identification of the double-bond positions of fatty acids, a portion of fatty acids were converted to picolinyl esters [73] to provide confirmatory fragmentation information. Polyunsaturated fatty acids and fatty acids in low (<10 ng) concentration were also validated by comparison of retention time and mass spectra of a 52-component fatty acid methyl ester standard (Nu-Chek Prep, Inc.). No copepods were analyzed for FA content.

Phytoplankton FA content per cell is presented as both absolute abundance (ng) and relative abundance (%) because *p*CO_2_ may affect cellular FA in two distinct ways: 1) *p*CO_2_ may alter the proportions of different EFAs in total intracellular FAs, which may affect crustacean physiological performance regardless of total EFA mass [55,74] or 2) *p*CO_2_ may alter the mass of intracellular EFA, which can affect zooplankton performance [38]. We defined PUFA as the sum of eleven PUFAs (see S3 Table for full list). We defined EFA as the sum of eicosapentaenoic acid (EPA), stearidonic acid (SDA), docosahexaenoic acid (DHA), and gamma-linolenic acid (GLA) (S3 Table).

Because phytoplankton species and their level of N deficiency used in the feeding mixtures differed between the two dates, the two experimental runs are not considered as true replicates and results are therefore presented separately (Table 1). FA data for *Isochyrsis galbana* was incomplete for the EFA classes of interest and therefore not included in the results or further analysis. Additionally, only some (~25% in Exp. 1and ~65% in Exp. II) of the 25 original female copepods were viable at the time of egg collection presumably due to natural causes as no instances of mishandling were known (Table 1).

### Data analysis

We did not perform statistical tests on phytoplankton FA data because only single monocultures of each species under each *p*CO_2_ treatment were used, hence no replicate samples were produced. Median values and interquartile ranges (as opposed to mean values) were chosen to describe the copepod reproductive parameters mainly due to skewness in the data. Poisson regressions were used to assess the effect of diet *p*CO_2_ treatment on egg production. Logistic regressions were used to assess the effects of diet *p*CO_2_ treatment on binary data, i.e., copepod hatching success and naupliar survival. The output of Poisson and logistic regression models is an odds ratio (OR), which predicts the odds of a certain outcome (i.e., hatching success relative to failure) under a specific treatment (i.e., high-*p*CO_2_ diet). Odds ratios equal to 1 indicate no effect of treatment on the outcome. Odds ratios with 95% confidence intervals that do not contain 1 are considered significant. Statistical tests were performed using R version 2.15.1 [75].

## Results

### Phytoplankton fatty acids

The absolute quantities of the various fatty acids (SFA, MUFA & PUFA) as well as the EFAs (sum of EPA,SDA,DHA,GLA) in the phytoplankton cultured and fed to the copepods during Experiment I were an order of magnitude less than in Experiment II (Tables 2 and 3). In Experiment I, the phytoplankton rarely fully depleted the ambient nitrate within the semi-continuous cultures prior to their daily dilution with fresh medium, whereas in Experiment II the ambient nitrate levels were consistently at or below the limit of analytical detection prior to replenishment, except for a higher nitrate concentration in the control *I*. *galbana* culture on Day 4 (S2 Table). These differences in N deficiency are reflected in the FA production attained by otherwise similarly maintained phytoplankton cultures.

**Table 2:** Experiment I (N-limited, December 2014) phytoplankton fatty acid concentrations.

FA class	*R. salina*			*S. marinoi*			*P. micans*			Mean	
	400 ppm	1,000 ppm	% difference	400 ppm	1,000 ppm	% difference	400 ppm	1,000 ppm	% difference	400 ppm	1,000 ppm
**EFA**	0.0044	0.0026	-41	0.00040	0.000090	-78	0.037	0.052	+41		
**Other PUFA**	0.0019	0.0012	-37	0.000071	0.000068	-4	0.003	0.023	+590		
**PUFA** (Sum of EFA, Other PUFA)	0.0063	0.0038	-40	0.00047	0.00016	-67	0.040	0.075	+88		
**SFA**	0.0054	0.0076	+40	0.00029	0.00011	-62	0.004	0.035	+870		
**MUFA**	0.0064	0.0055	-14	0.00019	0.00020	+5	0.013	0.093	+620		
**TFA** (Sum of SFA,MUFA,PUFA)	0.0181	0.0169	-6	0.00096	0.00047	-51	0.057	0.203	+260		
**EFA:PUFA**	70	68	—	91	58	—	91	69	—	84	65
**EFA:TFA**	19	11	—	30	13	—	37	12	—	29	12
**PUFA:TFA**	35	22	—	49	34	—	71	37	—	52	31

Absolute masses of the various fatty acid classes (ng cell^-1^) and relative contribution of essential fatty acids to polyunsaturated fatty acids (%), and essential fatty acids to total fatty acids (%) for each phytoplankton species under control (400 ppm) and high-*p*CO_2_ (1,000 ppm) treatments and the percentage change of fatty acid composition between treatments in Experiment I (December 2014). Fatty acid class abbreviations are: EFA–essential fatty acid; PUFA–polyunsaturated fatty acid; SFA–saturated fatty acid; MUFA–monounsaturated fatty acid; TFA–total fatty acids.

**Table 3:** Experiment II (N-depleted, June 2015) phytoplankton fatty acid concentrations.

FA class	*R*. *salina*			*S*. *marinoi*			*P*. *micans*			Mean	
	400 ppm	1,000 ppm	% difference	400 ppm	1,000 ppm	% difference	400 ppm	1,000 ppm	% difference	400 ppm	1,000 ppm
**EFA**	0.0417	0.0174	-58	0.0027	0.0033	+22	1.12	1.63	+46		
**Other PUFA**	0.0205	0.0147	-29	0.0012	0.0019	+58	0.14	0.21	+50		
**PUFA** (Sum of EFA, Other PUFA)	0.0622	0.0321	-48	0.0039	0.0052	+34	1.26	1.84	+47		
**SFA**	0.0999	0.0485	-65	0.0017	0.0058	+250	1.31	1.89	+44		
**MUFA**	0.0740	0.0826	+12	0.0059	0.0090	+51	0.44	0.67	+54		
**TFA** (Sum of SFA, MUFA, PUFA)	0.2362	0.1495	-38	0.0115	0.0200	+73	3.00	4.40	+47		
**EFA:PUFA**	67	54	—	69	63	—	89	89	—	75	69
**EFA:TFA**	17	11	—	23	15	—	36	37	—	25	21
**PUFA:TFA**	26	21	—	34	26	—	42	42	—	34	30

Absolute masses of the various fatty acid classes (ng cell^-1^) and relative contribution of essential fatty acids to polyunsaturated fatty acids (%), and essential fatty acids to total fatty acids (%) for each phytoplankton species under control (400 ppm) and high-*p*CO_2_ (1,000 ppm) treatments and the percentage change of fatty acid composition between treatments in Experiment II (June 2015). Fatty acid class abbreviations are: EFA–essential fatty acid; PUFA–polyunsaturated fatty acid; SFA–saturated fatty acid; MUFA–monounsaturated fatty acid; TFA–total fatty acids.

**Fig 2:**
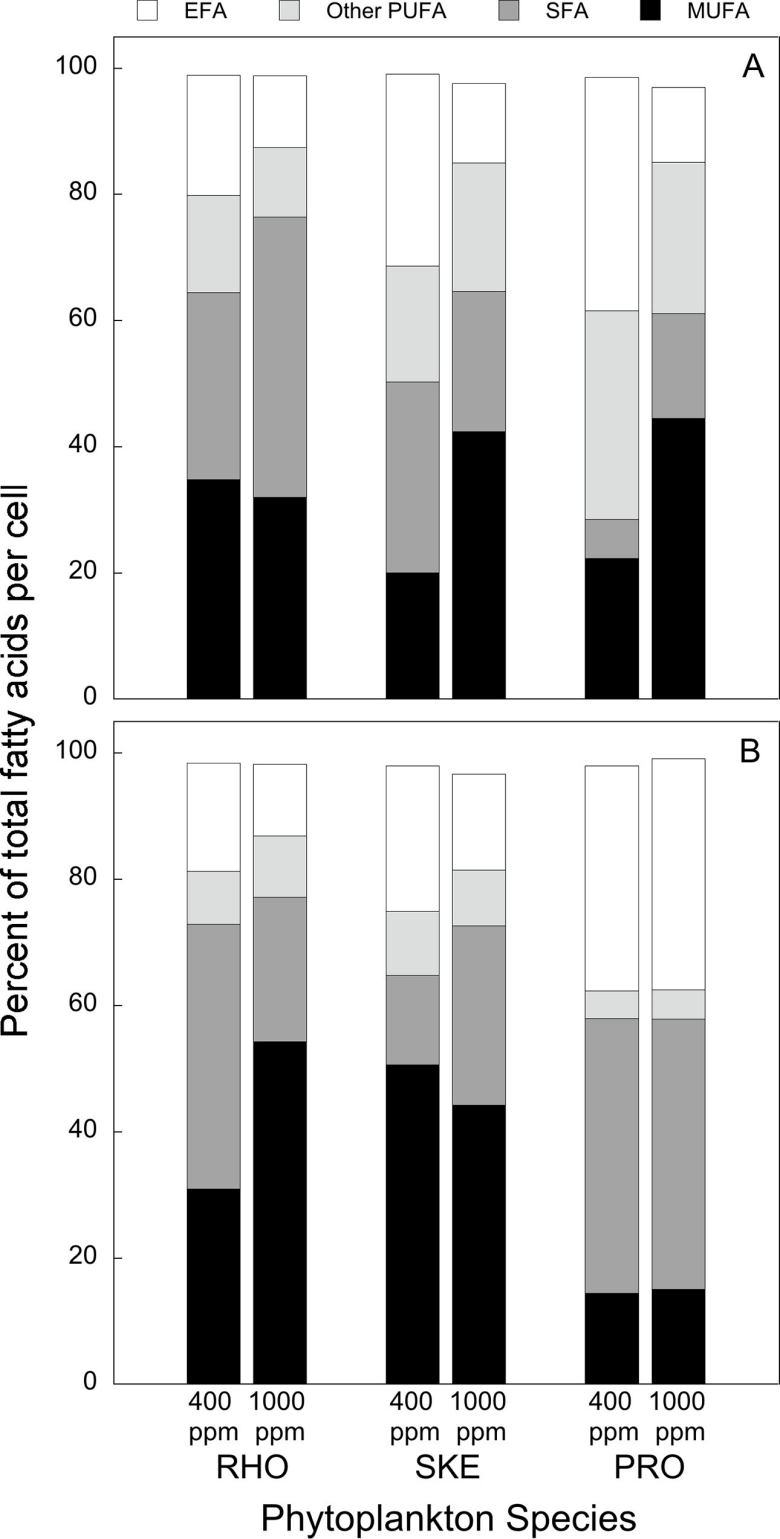
Phytoplankton fatty acid concentration. Percent essential fatty acid (EFA), other polyunsaturated fatty acid (PUFA), monounsaturated fatty acid (MUFA), and saturated fatty acid (SFA) comprising total fatty acid per cell under control (400 ppm) and high (1,000 ppm) *p*CO_2_ in Experiment I (A) and Experiment II (B).

In Experiment I, which was N-limited, the relative contributions of total PUFAs to total fatty acids (TFA) (PUFA:TFA) and total EFAs to total PUFAs (EFA:PUFA) were lower in the high-*p*CO_2_ treatment than in the control for the three species tested (*R*. *salina*, *S*. *marinoi*, and *P*. *micans*) (Fig 2A). The mean PUFA:TFA was higher in the control (52%) than in high *p*CO_2_ (31%), and the same trend was seen for mean EFA:PUFA (84% in control and 65% in high *p*CO_2_) (Table 2). The absolute cell contents of total PUFA and EFA in *R*. *salina* were 40 and 41% lower, respectively, in high *p*CO_2_ than in the control (Table 2). Similarly, *in S*. *marinoi* the absolute cell contents of total PUFA and EFA were 67 and 78% lower in high *p*CO_2_ treatment than the control, respectively (Table 2). On the other hand, cell contents of total PUFA and EFA of *P*. *micans* were 88 and 41% greater in high *p*CO_2_ treatment than in the control, respectively (Table 2).

Dietary FAs from *Isochrysis galbana* were not quantified in Experiment II (Table 1). Therefore, the dietary FAs presented for Experiment II represent only ~75% of the FAs fed to copepods during that experiment. In Experiment II, which was N-depleted, the relative contributions of PUFAs and EFAs to total FAs were lower in the high-*p*CO_2_ treatment only for *R*. *salina* and *S*. *marinoi* (Fig 2B). Across all species, the mean PUFA:TFA was higher in the control (34%) than in high *p*CO_2_ (30%), and the same trend was seen for the mean EFA:PUFA (75% in control and 69% in high *p*CO_2_) (Table 3). The absolute cell contents of total PUFA and EFA of *R*. *salina* were 48 and 58% lower in high *p*CO_2_ than in the control (Table 3). However, in *S*. *marinoi* the absolute cell contents of total PUFA and EFA were 34 and 22% greater in high *p*CO_2_ than in the control (Table 3). Absolute total PUFA and EFA cell contents were also greater in high *p*CO_2_ than in the control in *P*. *micans* (47 and 46%, respectively) (Table 3).

We saw similar trends in the overall FA masses and class proportions of the diets fed to the copepods based on cellular FA concentration and the cell abundance of each phytoplankton species in these diets. In Experiment I, copepods were fed less EFA under high *p*CO_2_ (21.82 μg L^-1^) than under control *p*CO_2_ (34.84 μg L^-1^), but equal quantities of total fatty acids (Table 4). In this experiment, the high-*p*CO_2_ copepod diet also contained less total PUFA (32.04 μg L^-1^) compared to the control diet (48.30 μg L^-1^) (Table 4). Similar relative differences in EFAs and PUFAs were measured in Experiment II, in which the copepod diet contained less EFA (194.40 μg L^-1^) and less total PUFA (289.56 μg L^-1^) under high *p*CO_2_ than under control *p*CO_2_ (280.88 μg L^-1^ and 396.60 μg L^-1^, respectively) (Table 5) but in contrast to Experiment I, total fatty acids Experiment II were 21% lower in the high-*p*CO_2_ treatment than in the control.

**Table 4:** Fatty acid concentrations in copepod diets, Experiment I (N-limited, December 2014).

FA class	*R*. *salina*		*S*. *marinoi*		*P*. *micans*		Total	
	400 ppm	1,000 ppm	400 ppm	1,000 ppm	400 ppm	1,000 ppm	400 ppm	1,000 ppm
**EFA**	29.50	17.45	2.67	0.59	2.67	3.78	34.84	21.82
**Other PUFA**	12.74	8.05	0.47	0.46	0.25	1.71	13.46	10.22
**PUFA** (Sum of EFA, Other PUFA)	42.24	25.5	3.14	1.05	2.92	5.49	48.30	32.04
**SFA**	36.52	51.22	1.95	0.69	0.26	2.54	38.73	54.45
**MUFA**	42.79	36.91	1.29	1.32	0.94	6.84	45.02	45.07
**TFA** (Sum of SFA, MUFA, PUFA)	121.54	113.64	6.38	3.04	4.12	14.88	132.04	131.56

Absolute masses of fatty acids fed to copepods (μg C per 1-L feeding chamber) in Experiment I (December 2014) based on the cellular fatty acid content and the number of each phytoplankton species included in the feeding mixture. Fatty acid class abbreviations are: EFA–essential fatty acid; PUFA–polyunsaturated fatty acid; SFA–saturated fatty acid; MUFA–monounsaturated fatty acid; TFA–total fatty acids.

**Table 5:** Fatty acid concentrations in copepod diets, Experiment II (N-depleted, June 2015).

FA class	*R*. *salina*		*S*. *marinoi*		*P*. *micans*		Total	
	400 ppm	1,000 ppm	400 ppm	1,000 ppm	400 ppm	1,000 ppm	400 ppm	1,000 ppm
**EFA**	208.58	88.17	13.43	16.34	58.87	89.89	280.88	194.40
**Other PUFA**	102.59	74.45	5.90	9.43	7.23	11.28	115.72	95.16
**PUFA** (Sum of EFA, Other PUFA)	311.17	162.62	19.33	25.77	66.10	101.17	396.60	289.56
**SFA**	499.96	176.91	8.18	28.43	69.36	104.12	577.50	309.16
**MUFA**	370.04	418.85	29.44	44.40	22.95	36.78	422.43	500.03
**TFA** (Sum of SFA, MUFA, PUFA)	1,181.17	758.38	56.96	98.60	158.42	242.07	1,395.56	1,099.05

Absolute masses of fatty acids fed to copepods (μg C per 1-L feeding chamber) in Experiment II (June 2015) based on the cellular fatty acid content and the number of each phytoplankton species included in the feeding mixture. Fatty acid class abbreviations are: EFA–essential fatty acid; PUFA–polyunsaturated fatty acid; SFA–saturated fatty acid; MUFA–monounsaturated fatty acid; TFA–total fatty acids.

### Copepod reproductive success

**Fig 3:**
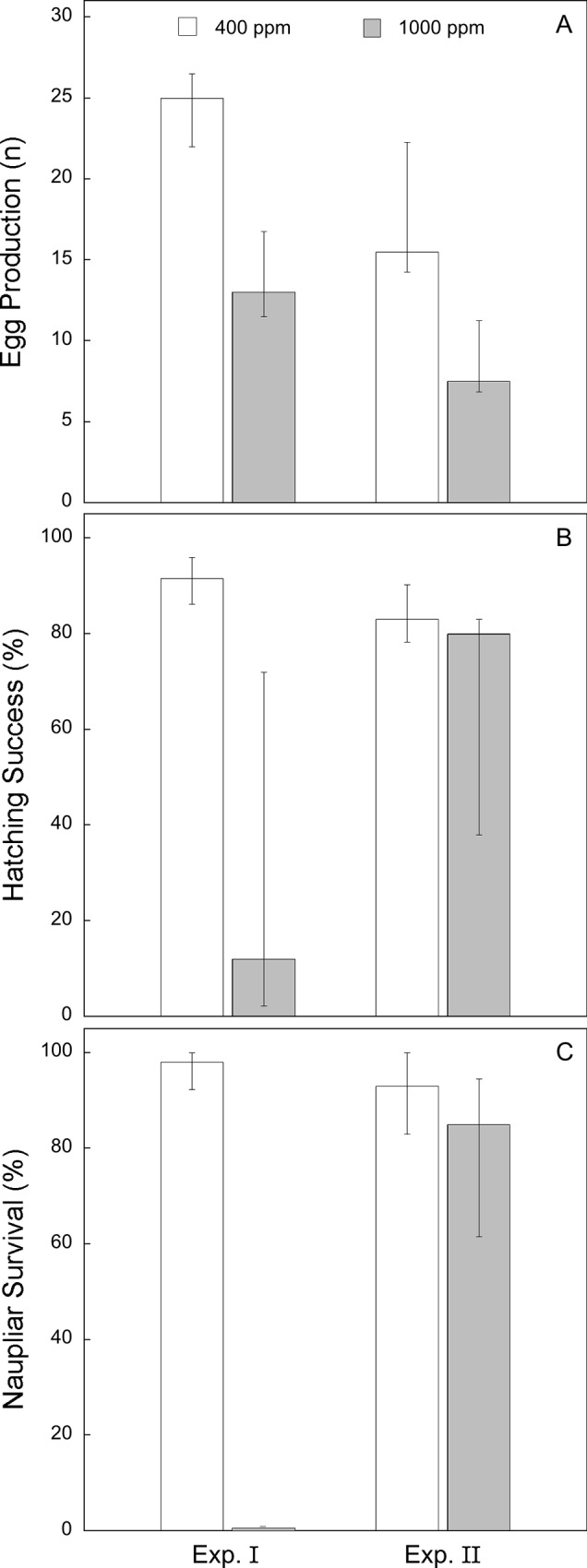
Copepod reproductive success. Copepod egg production rate (eggs female^-1^d^-1^) (A), percent hatching success (B), and naupliar survival (C) in Experiment I and II after being fed control (400 ppm) and high (1,000 ppm) *p*CO_2_ diets. Bar heights represent median values female^-1^ for all reproductive parameters. Error bars represent interquartile ranges.

In both experiments, the median values of the three reproductive parameters, egg production, hatching success, and naupliar survival, were lower in the copepods fed high-*p*CO_2_ phytoplankton than for copepods fed phytoplankton from the control (Fig 3). In N-limited Experiment I, the copepods fed phytoplankton diets from the high-*p*CO_2_ treatment had median values of egg production, hatching success, and naupliar survival that were 48%, 87%, and 100% lower, respectively, than copepods in controls. In N-depleted Experiment II, median values of egg production, hatching success, and naupliar survival were 52%, 4%, and 9% lower, respectively, in copepods fed high-*p*CO_2_ phytoplankton than in copepods fed control phytoplankton.

Poisson (used for egg production) and logistic (used for hatching success and naupliar survival) regression analyses demonstrate that dietary *p*CO_2_ condition significantly affected all three reproductive parameter outcomes in both experiments. The odds ratios and their upper confidence limits were all less than one, giving high confidence that high *p*CO_2_ reduced egg production as well as the likelihood of egg hatching success and naupliar survival (Table 6).

**Table 6:** Copepod reproductive success.

		Egg production	Hatching success	Naupliar survival
**Experiment I**	**Log odds ratio**	0.61	0.02	0.01
	**95% CI**	(0.47, 0.79)	(0.01, 0.05)	(0.00, 0.04)
**Experiment II**	**Log odds ratio**	0.45	0.55	0.15
	**95% CI**	(0.36, 0.56)	(0.42, 0.77)	(0.08, 0.27)

Log odds ratios and 95% confidence intervals (CI) from Poisson regressions (egg production) and logistic regressions (hatching success and naupliar survival). LOR <1 and confidence intervals that do not contain 1 indicate that *p*CO_2_ treatment had a significant negative effect on the reproductive parameter.

## Discussion

Our experiment was designed to assess changes in copepod reproductive success resulting from *p*CO_2_-induced changes in the FA composition of the phytoplankton diet consumed by the copepods, since direct effects of OA on copepod reproduction are unlikely [14,18,76]. We conducted the experiments within the context of N-deficiency due to the N-limited nature of most coastal marine systems and the impact of N availability on phytoplankton FA. The results from this laboratory study demonstrate that high *p*CO_2_ does not necessarily reduce FA synthesis, but it increases FA saturation (i.e., lower relative PUFA content) thereby affecting the EFA content of three phytoplankton species tested here. Furthermore, the results show that N-depletion (as opposed to N-limitation) increased FA synthesis among all FA groups and phytoplankton species tested under both low and high *p*CO_2_. We suggest that the CO_2_-induced changes in the nutritional quality of a copepod’s diet seen here impaired copepod reproductive success, but the increase in FA under N-depleted conditions reduced the negative effect on hatching success and naupliar survival. Our findings are consistent with the main finding of Rossoll et al. [33] in which only one prey species was used.

However, the effect of OA on the FA concentration and FA composition of phytoplankton varies by species since not all of the phytoplankton species used in this study responded similarly to high *p*CO_2_. In N-limited Experiment I for example, the intracellular masses of EFA in *R*. *salina* and *S*. *marinoi* were lower under high *p*CO_2_ than under control *p*CO_2_, but *P*. *micans* had the opposite response (Table 2). The different FA responses seen for *S*. *marinoi* and *T*. *pseudonana* between this study and Rossoll et al. [33] are likely examples of the variable taxonomic responses of diatoms to high *p*CO_2_ suggested by studies that report lower [33], higher [56,57], or no change [36] in EFA under high *p*CO_2_. Similar EFA variability in response to high *p*CO_2_ has been documented also in green algae [62,77–79]. While changes in the absolute contents of cellular FAs varied between experiments and among species, and even within the same species in the two experiments (e.g., *S*. *marinoi*), cellular EFA:PUFA was consistently lower under high *p*CO_2_ for all species (Tables 2 and 3). PUFA:TFA in each cell was also consistently lower under high *p*CO_2_, except for that of *P*. *micans* in Experiment II (Table 3). Furthermore, the masses of both EFAs and PUFAs in the mixed diet of phytoplankton fed to the copepods were less in the high-*p*CO_2_ treatments of both experiments (Tables 4 and 5).

The absolute cellular FA contents varied substantially between the two experiments (Tables 4 and 5). Masses of nearly all the FA classes for the three phytoplankton species under both *p*CO_2_ treatments were an order of magnitude higher in Experiment II than in Experiment I. We attribute this mass disparity to differences in the level of N deficiency of the unialgal cultures used for the two experiments (S2 Table). Whereas fully N-depleted cultures were used in the dietary mixture in Experiment II (with the exception of control-*p*CO_2_ *I*. *galbana*), the semi-continuous cultures in Experiment I can only be considered N-limited (with the exception of high-*p*CO_2_ *P*. *micans*) since measurable nitrate concentrations were routinely found prior to their daily dilution with fresh media. The relationship between nutrient deficiency and the production of lipids and FAs has long been known, with generally increasing lipid production rates as macronutrients, particularly N, become scarce and then fully depleted, although the relationship between lipid content and nutrient limitation is species-specific [8–83].

Combining observations of phytoplankton FA with those of the copepod reproductive response, two patterns emerge in the results of the two experiments: 1) reproductive success was depressed when copepods were fed a high-*p*CO_2_ phytoplankton diet, and 2) this depression varied between Experiment I and Experiment II. First, the significant declines in copepod reproductive output when fed the high-*p*CO_2_ phytoplankton diets of lower EFA:PUFA and PUFA:TFA may suggest that overall copepod reproductive response was more influenced by EFA and PUFA proportions than absolute masses of FA. Second, the inconsistent trend in the severity of the decline in reproductive output under a high-*p*CO_2_ diet (more severe in Experiment I) compared to the absolute masses of EFAs and PUFAs (an order of magnitude lower in Experiment I) may indicate that the intensity of the copepod response was more influenced by the absolute mass of dietary EFAs and PUFAs than by proportions of FA classes.

Each measure of copepod fecundity declined when copepods were fed a high-*p*CO_2_ diet (Fig 3), but these decreases were more severe in the Experiment I than in Experiment II, likely due to higher concentrations of FA in Experiment II (N-depleted) [44–51]. The product of these three measures (egg production, hatching success, and naupliar survival) gives a measure of the overall median reproductive success female^-1^. In Experiment I, the product equaled zero viable nauplii female^-1^ under the high-*p*CO_2_ diet, whereas the product equaled 22 viable nauplii female^-1^ under the control diet. An overall 0% survival presents an obvious problem if this were to occur in a natural population, though the low number of copepods used in Experiment I (six females for each treatment) limits the scope of inference for this part of the study. In Experiment II, copepods fed the high-*p*CO_2_ diet produced a median of 5 viable nauplii female^-1^, compared to the median of 19 viable nauplii female^-1^ produced by copepods fed the control diet (16 females for each treatment). Taken together, these experimental results indicate reductions in reproductive success that would be devastating to most natural populations.

In comparing reproductive responses between experiments, both environmental and genetic variables can affect copepod reproduction and may explain differences seen in the reproductive responses between the two experiments. In natural environments, seasonal differences such as temperature, salinity, and food availability affect aspects of copepod reproduction [84,85] and could account for differences seen between the two experiments. Differences in female growth and feeding history, male robustness, phenotypes within a species, and age of females that reproduced in the experiments also could have contributed to the difference in response between Experiments I and II. Furthermore, our test zooplankter–*A*. *tonsa*–is probably a cryptic species complex [86,87], meaning populations identified as *A*. *tonsa* around the world may actually be different species. Therefore, genetic differences between the copepods collected in December 2014 (Exp. I) and June 2015 (Exp. II) may have influenced fecundity [88]. In addition, only three species (*R*. *salina*, *S*. *marinoi*, and *P*. *micans*) were used in the feeding mixtures for Experiment I, whereas *I*. *galbana* was added to the dietary mixture (Table 1) for Experiment II, although its FA content could not be assessed. Though the total carbon content of the feeding mixture was the same in both experiments, the different phytoplankton dietary mixtures may have affected reproductive success [89].

The responses of copepod reproduction to different conditions, including OA, may differ in their mean, variance, or both. Results from our study (Fig 3) show an effect on variance, possibly indicating high genetic variation in responses among individuals within a population, although the small number of copepods in Experiment I limits conclusions about those results. Copepod hatching success (both experiments) and naupliar survival (Experiment II) varied more widely under the high-*p*CO_2_ treatment than under control *p*CO_2_ for each respective parameter (Fig 3B and 3C). This variability in could be due to dietary and environmental factors mentioned above but may also be an indication of natural variability in wild *A*. *tonsa* populations. This natural variability may allow *A*. *tonsa* populations in San Francisco Bay to adapt to OA-induced changes in the environment or food supply and there is evidence of this ecophysiological variability in copepod reproduction and metabolism [21,90,91]. Because OA will affect all individuals of a population, revealing variability in responses is essential for understanding how OA will affect populations and how those effects will cascade through trophic levels.

The availability of nutritious food under OA is likely crucial for copepods to function as an important trophic link in marine food webs. Copepods have developed a number of strategies to cope with risk of predation (e.g., high fecundity and elevated growth rates, limited motion, strong escape responses, low respiration rates, and extended reproductive viability), and many depend on the availability and consumption of high-quality food [92]. Given that copepods can change their feeding behavior in response to environmental conditions including food quality [48,52,90], it is possible that the copepod feeding behavior in our study differed between the two *p*CO_2_ treatments and thereby influenced copepod reproductive success [52,89]. Poor food quality or food deprivation may not only limit copepod reproduction but may also exacerbate the negative effects of OA on metabolic functions [16,93,94], further impairing copepod productivity. We also note that excess or surplus carbon from increased CO_2_ in the experimental system could have affected copepod metabolism and therefore copepod reproduction. If copepods feeding on the high-*p*CO_2_ phytoplankton diet ingested excess carbon compared to those feeding on the control phytoplankton, they would have needed to either make use of or dispose of excess ingested C, which could have shifted metabolic functions including those linked to egg production [95–97]. We also must note that OA conditions often increase the C:N ratio of phytoplankton, thereby decreasing their nutritional quality for consumers [53]. Although we did not measure the elemental composition (particulate C, N, and P) of the phytoplankton or copepods in this study, such biochemical changes in addition to FA could have also influenced the reproductive responses seen here.

In addition to directly affecting phytoplankton nutritional quality at the cellular level, high *p*CO_2_ also drives changes in the phytoplankton species composition of natural assemblages. Changes in the FA composition of future phytoplankton assemblages will likely be due to shifts in both growth conditions and community composition, including taxonomic and size distributions [38,39,98]. The dominant size fraction in phytoplankton assemblages may shift from the nano-size fraction (2.7–10 μm) to the pico-size fraction (0.3–2.7μm), which are less available to copepods because of their smaller size [96] and are less nutritious due to their lower proportion of PUFAs [38]. In some situations, *p*CO_2_-driven changes in community composition may actually compensate for negative effects of *p*CO_2_ on cellular FA [98], but this finding has certainly not been universal [38].

The changes in phytoplankton EFA seen in this study also have implications for the productivity of other herbivorous zooplankton and higher trophic levels. Many studies have shown the positive influence of phytoplankton omega-3 FAs and other EFA on growth and reproduction of freshwater cladocerans [99–102]. High EFA concentrations of phytoplankton and copepod eggs improved the conditions of cod [103] and herring [104] larvae. Additionally, abnormally high ratios of ω-3 to ω-6 fatty acids have been found in phytoplankton assemblages in regions where the M74 syndrome is prevalent (a reproductive disturbance that can cause up to 90% mortality in salmon larvae) [105]. Although the changes in phytoplankton EFA mentioned in these previous studies were not attributed to OA, the results of our study support the hypothesis that future OA-conditions will reduce the quality of trophic transfer and negatively affect higher trophic levels.

In contrast to our study, the results from another similar study using *A*. *grani* as consumer and the dinoflagellate *Heterocapsa* sp. as prey showed no negative effects of OA on FA content of algae nor on the fecundity of female copepods [37]. The inconsistent results among studies similar to ours [33,37] suggest the indirect effects of OA on copepod reproduction depend upon the prey. These differences emphasize the species-specific responses of phytoplankton FA to OA and the importance of considering the mixed structure of natural copepod diets.

Recent studies on the combined direct effects of changing environmental conditions such as increased temperature and decreased pH on planktonic FA provide diverse results, but many of the results do not account for potential indirect dietary effects. The EFA content of some copepods were more sensitive to warming than to acidification [106], while other copepods showed an overall decrease in PUFAs, including EFAs, under increased acidification conditions [107]. Our findings along with other reports of phytoplankton EFA sensitivity to warming [108] and OA [33], suggest that indirect effects of OA through the diets of copepods should be as much a focus of research as direct effects.

## Conclusions

The copepod *A*. *tonsa* showed impaired reproductive ability when fed a diet of high-*p*CO_2_ phytoplankton with reduced EFA and PUFA, suggesting that fecundity of *A*. *tonsa* may decrease under future OA conditions. Based on our results using *R*. *salina* and *S*. *marinoi*, cryptophytes and diatoms were more susceptible to decreases in their cellular EFA content due to increasing *p*CO_2_, whereas the response for the tested dinoflagellate *P*. *micans* varied between the two experiments. OA-induced changes in phytoplankton EFA content appears to be species-dependent and may inhibit copepod productivity through altering the lipids available for copepod egg production, egg hatching success, and naupliar survival. Most previous studies that have investigated the direct effects of OA on copepods have found copepods to be widely resilient to future-predicted OA conditions, whereas our study demonstrates that copepods were indirectly affected by OA through changes in the availability of EFAs in their planktonic diets.

## Supporting information

S1 Table Phytoplankton culture pH measurements. pH of low *p*CO_2_ (400 ppm) and high *p*CO_2_ (1,000 ppm) phytoplankton feeding mixtures fed to copepods during the two four-day experiments. T_0_ and T_f_ measurements are those taken immediately before and after the 24-h incubation period.
(DOCX)

S2 Table Phytoplankton culture nutrient measurements. Nitrate plus nitrite (NO_3_ + NO_2_) concentrations (μM) of the low *p*CO_2_ (400 ppm) and high *p*CO_2_ (1,000 ppm) phytoplankton monocultures on days 1–4 of the two experiments. Samples were collected immediately prior to daily culture dilution with fresh medium. 0.0 = concentration below the detection limit of 0.04 μM for nitrate + nitrite analysis.
(DOCX)

S3 Table List of polyunsaturated fatty acids (PUFA) and essential fatty acids (EFA) included in analysis. *Indicates EFA.
(DOCX)
