# Supplementary material for: Effect of ocean acidification on the nutritional quality of marine phytoplankton for copepod reproduction
Source: PLoS One. 2019 May 20;14(5):e0217047. doi: 10.1371/journal.pone.0217047 (PMC6527307; doi:10.1371/journal.pone.0217047)
Supplement: S1 Table — pH of low pCO2 (400 ppm) and high pCO2 (1,000 ppm) phytoplankton feeding mixtures fed to copepods during the two four-day experiments. T0 and Tf measurements are those taken immediately before and after the 24-h incubation period. (DOCX) [file pone.0217047.s001.docx]

|  | **Experiment I (December 2014)** | | | | **Experiment II (June 2015)** | | | |
| --- | --- | --- | --- | --- | --- | --- | --- | --- |
|  | 400 ppm | | 1,000 ppm | | 400 ppm | | 1,000 ppm | |
| **Day** | T_0_ | T_f_ | T_0_ | T_f_ | T_0_ | T_f_ | T_0_ | T_f_ |
| **1** | 8.12 | 8.11 | 7.81 | 7.82 | 8.11 | 8.17 | 7.83 | 7.80 |
| **2** | 8.16 | 8.13 | 7.85 | 7.85 | 8.19 | 8.08 | 7.88 | 7.76 |
| **3** | 8.16 | 8.12 | 7.89 | 7.88 | 8.16 | 8.09 | 7.84 | 7.79 |
| **4** | 8.18 | 8.14 | 7.87 | 7.83 | 8.18 | 8.16 | 7.91 | 7.88 |

**S1 Table**
