# Supplementary material for: Effect of ocean acidification on the nutritional quality of marine phytoplankton for copepod reproduction
Source: PLoS One. 2019 May 20;14(5):e0217047. doi: 10.1371/journal.pone.0217047 (PMC6527307; doi:10.1371/journal.pone.0217047)
Supplement: S2 Table — Nitrate plus nitrite (NO3 + NO2) concentrations (μM) of the low pCO2 (400 ppm) and high pCO2 (1,000 ppm) phytoplankton monocultures on days 1–4 of the two experiments. Samples were collected immediately prior to daily culture dilution with fresh medium. 0.0 = concentration below the detection limit of 0.04 μM for nitrate + nitrite analysis. (DOCX) [file pone.0217047.s002.docx]

|  | Experiment I (December 2014) | | | | | | Experiment II (June 2015) | | | | | | | |
| --- | --- | --- | --- | --- | --- | --- | --- | --- | --- | --- | --- | --- | --- | --- |
|  | *R. salina* | | *S. marinoi* | | *P. micans* | | *R. salina* | | *S. marinoi* | | *P. micans* | | *I. galbana* | |
| Day | 400  ppm | 1,000 ppm | 400 ppm | 1,000  ppm | 400 ppm | 1,000  ppm | 400 ppm | 1,000  ppm | 400  ppm | 1,000 ppm | 400  ppm | 1,000 ppm | 400  ppm | 1,000  ppm |
| 1 | 0.00 | 0.37 | 2.84 | 0.00 | 0.55 | 18.46 | 0.00 | 0.00 | 0.00 | 0.00 | 0.00 | 0.00 | 0.04 | 0.00 |
| 2 | 0.00 | 0.28 | 1.15 | 0.00 | 1.61 | 17.77 | 0.00 | 0.00 | 0.00 | 0.00 | 0.00 | 0.00 | 0.10 | 0.07 |
| 3 | 0.55 | 3.67 | 1.60 | 1.12 | 2.43 | 18.0 | 0.07 | 0.04 | 0.00 | 0.05 | 0.00 | 0.07 | 0.20 | 0.12 |
| 4 | 1.52 | 2.46 | 0.92 | 0.95 | 0.78 | 15.15 | 0.24 | 0.13 | 0.00 | 0.11 | 0.07 | 0.00 | 3.01 | 0.18 |

**S2 Table.**
