# Supplementary material for: Effect of ocean acidification on the nutritional quality of marine phytoplankton for copepod reproduction
Source: PLoS One. 2019 May 20;14(5):e0217047. doi: 10.1371/journal.pone.0217047 (PMC6527307; doi:10.1371/journal.pone.0217047)
Supplement: S3 Table — *Indicates EFA. (DOCX) [file pone.0217047.s003.docx]

**
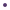
S3 Table**

| **PUFA** | **EFA** |
| --- | --- |
| C16:2 (multiple isomers summed, bond position could not be verified) |  |
| C16:3(n-4) |  |
| C16:x PUFA |  |
| C18:2(n-6) |  |
| C18:3(n-6)* | Gamma Linolenic Acid (GLA) |
| C18:4(n-3)* | Stearidonic Acid (SDA) |
| C20:4(n-6) |  |
| C20:5(n-3)* | Eicosapentaenoic Acid (EPA) |
| C22:5(n-6) |  |
| C22:6(n-3)* | Docosahexaenoic Acid (DHA) |
| VHUFA (very highly unsaturated fatty acid) |  |
